# Supplementary material for: Coumarin-Chalcone Hybrids as Inhibitors of MAO-B: Biological Activity and In Silico Studies
Source: Molecules. 2021 Apr 22;26(9):2430. doi: 10.3390/molecules26092430 (PMC8122463; doi:10.3390/molecules26092430)
Supplement: Supplementary file 1 [file molecules-26-02430-s001.zip › molecules-1187731-supplementary.pdf]

# Supplementary Materials

# Coumarin-Chalcone Hybrids as Inhibitors of MAO-B: Biological Activity and In Silico Studies

**Guillermo Moya-Alvarado [1,†], Osvaldo Yañez [2,3,†], Nicole Morales [4], Angélica González-González [5], Carlos Areche [6], Marco Tulio Núñez [7], Angélica Fierro [8,*] and Olimpo García-Beltrán [9,10,*]**

[1] Biology Department, Johns Hopkins University, Baltimore, MD 21218, USA; gmoya@bio.puc.cl
[2] Center of New Drugs for Hypertension (CENDHY), Santiago 8330015, Chile; osvyanezosses@gmail.com
[3] Computational and Theoretical Chemistry Group, Departamento de Ciencias Químicas, Facultad de Ciencias Exactas, Universidad Andres Bello, República 498, Santiago 7550196, Chile; osvyanezosses@gmail.com
[4] Department of Physiology, Faculty of Biological Sciences, Pontificia Universidad Católica de Chile, Santiago 8331150, Chile; nlmorales@uc.cl
[5] Laboratorio de Interacciones Insecto-Planta, Instituto de Ciencias Biológicas, Universidad de Talca, Casilla 747, Talca 3460000, Chile; angelica.gonzalez@utalca.cl
[6] Department of Chemistry, Faculty of Sciences, Universidad de Chile, Las Palmeras 3425, Nuñoa, 7800024 Santiago, Chile; areche@uchile.cl
[7] Biology Department, Faculty of Sciences, Universidad de Chile, Santiago 7800024, Chile; mnunez@uchile.cl
[8] Department of Organic Chemistry, Faculty of Chemistry, Pontificia Universidad Católica de Chile, Casilla 306, Santiago 6094411, Chile
[9] Universidad Bernardo O'Higgins, Centro Integrativo de Biología y Química Aplicada (CIBQA), General Gana 1702, Santiago 8370854, Chile
[10] Facultad de Ciencias Naturales y Matemáticas, Universidad de Ibagué, Carrera 22 Calle 67, Ibagué 730002, Colombia
† These authors contributed equally to this work
* Correspondence: afierroh@uc.cl (A.F.); jose.garcia@unibague.edu.co (O.G.-B.)

*Synthetic route*

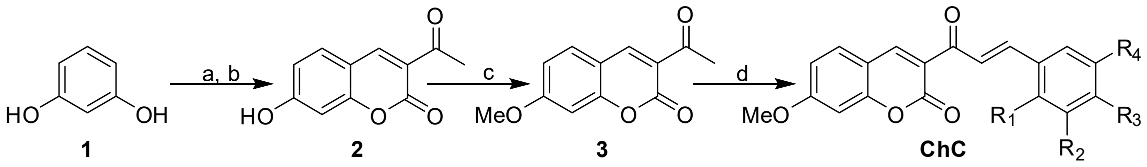


Scheme S**1**. Synthetic route to compounds **ChC1–ChC14**. Reagents and conditions: a) $POCl_3$, DMF, acetonitrile, 0-5 °C, 2 h, 88%; b) ethyl acetoacetate, ethanol, reflux, 6 h, 70%; c) $Me_2SO_4$, $K_2CO_3$, DMF, $H_2O$, 1 h, 78%; d) benzaldehyde, piperidine, DCM, reflux, 8 h, 25-47%.

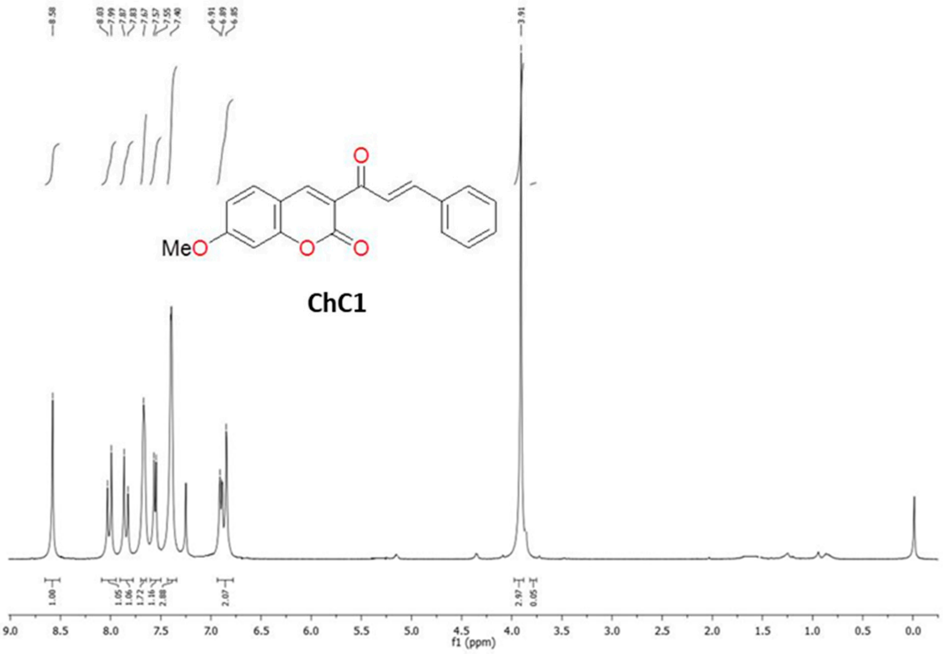


**Figure S1:** $^1$H NMR spectrum of **ChC1**.

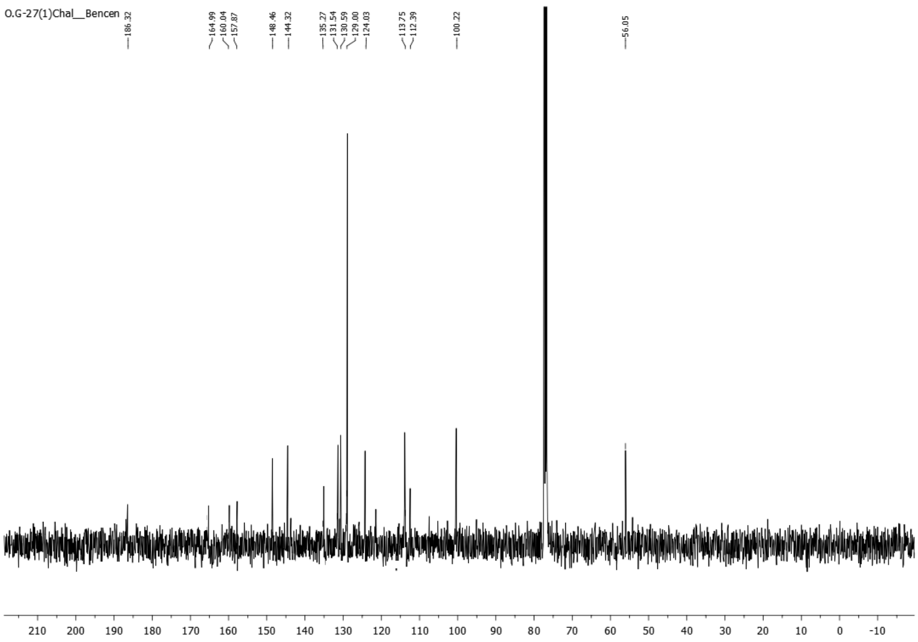


**Figure S2:** $^{13}$C NMR spectrum of **ChC1**.

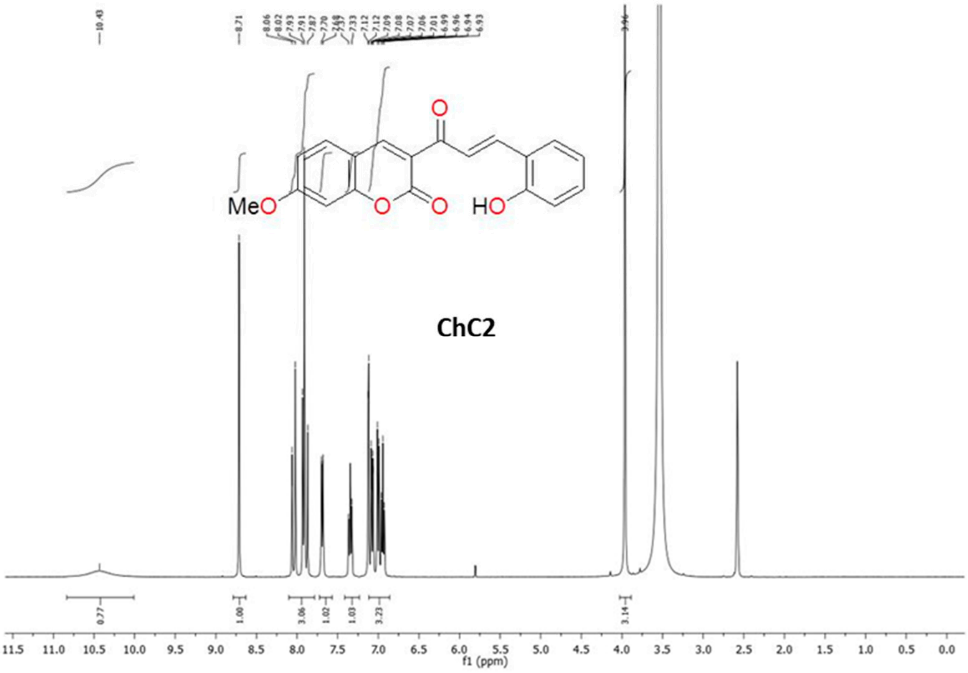


**Figure S3:** $^{1}$H NMR spectrum of **ChC2**.

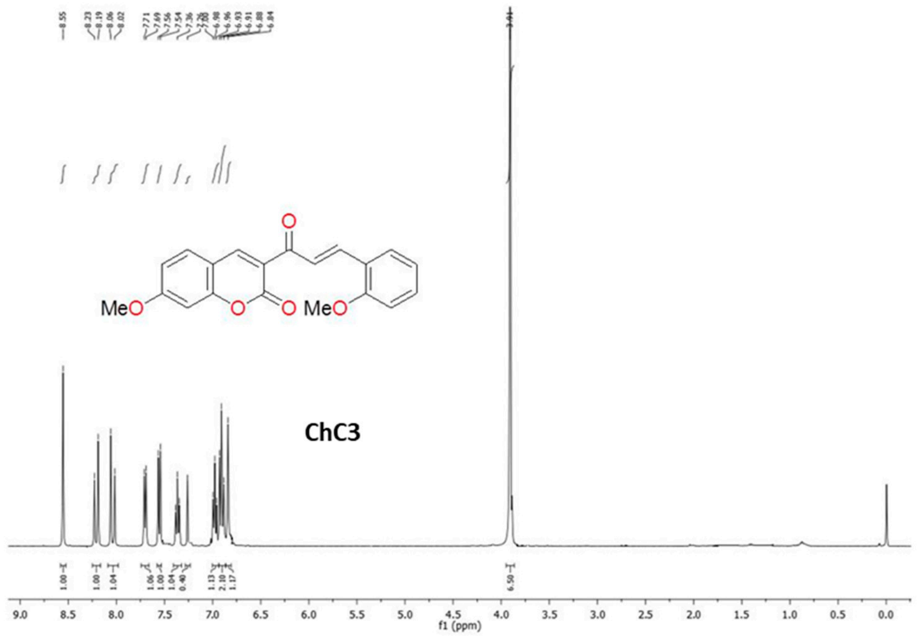

**Figure S4**. $^{1}$H NMR spectrum of **ChC3**.

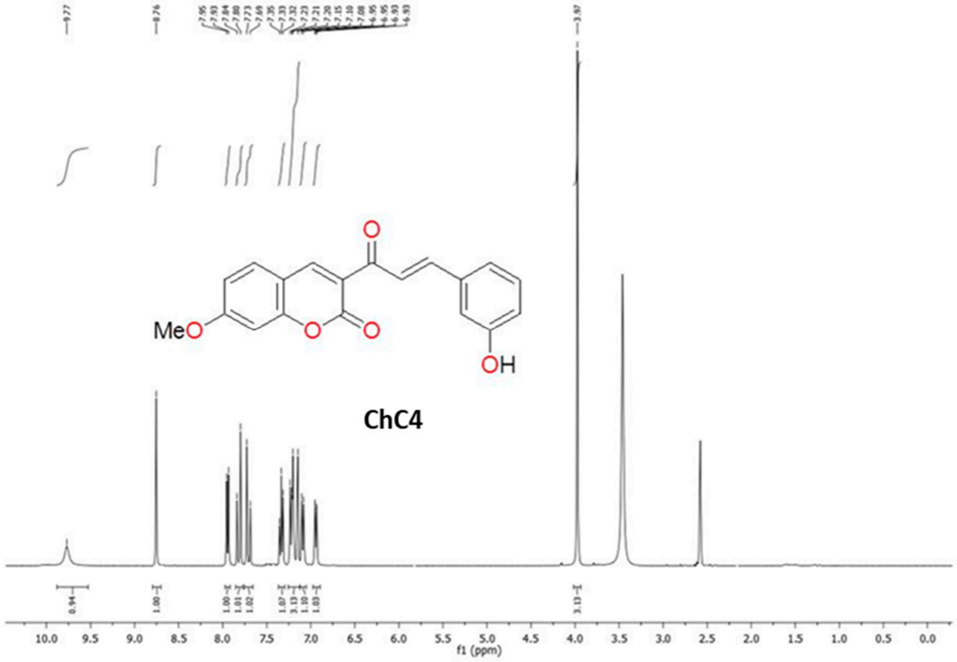

**Figure S5**. $^{1}$H NMR spectrum of **ChC4**.

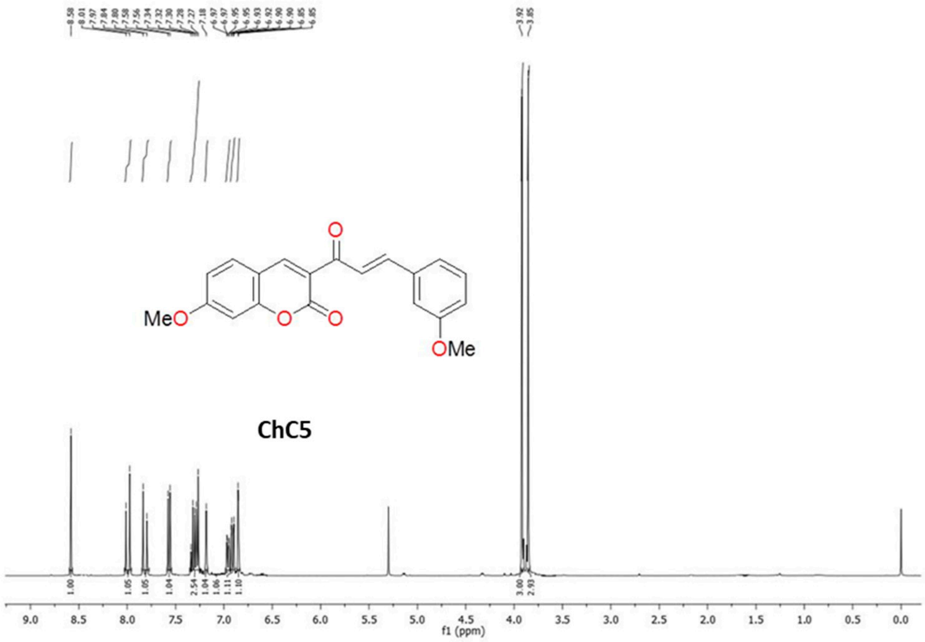


**Figure S6**. $^{1}$H NMR spectrum of **ChC5**.

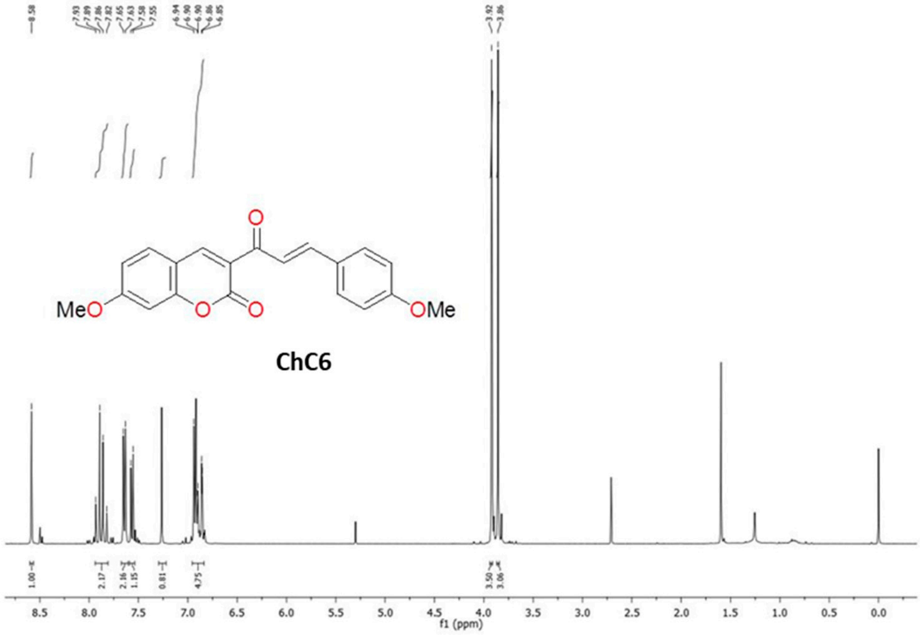


**Figure S7**. $^{1}$H NMR spectrum of **ChC6**.

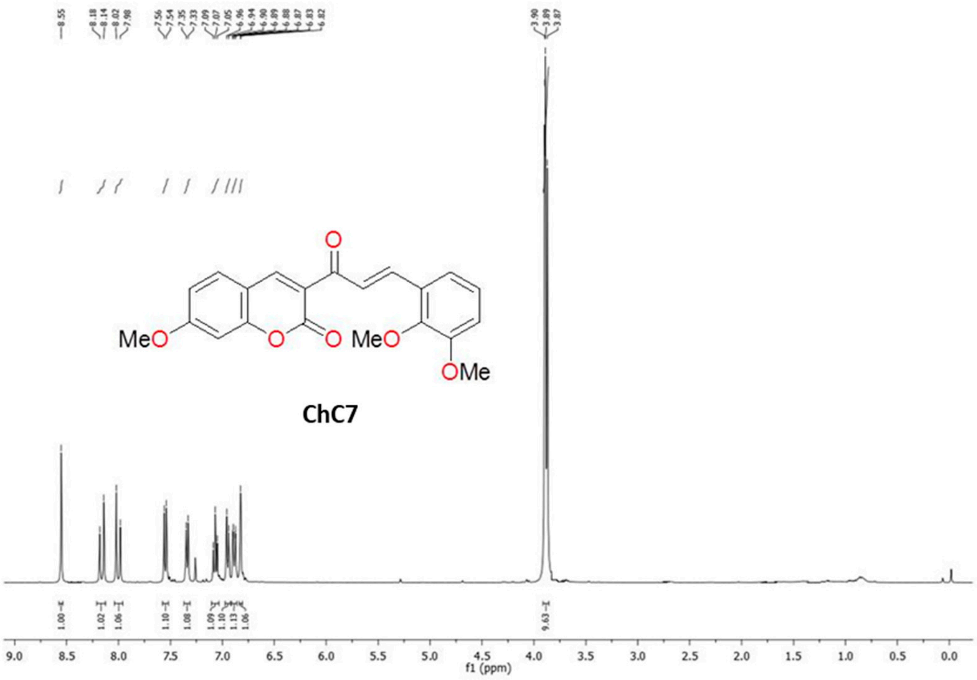


Figure S8. $^{1}$H NMR spectrum of **ChC7**.

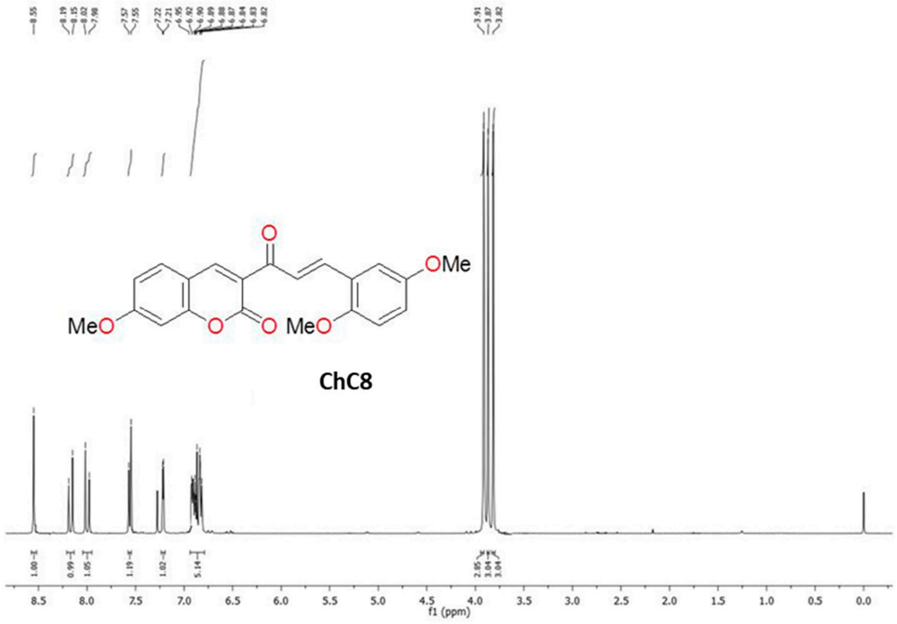


**Figure S9**. $^{1}$H NMR spectrum of **ChC8**.

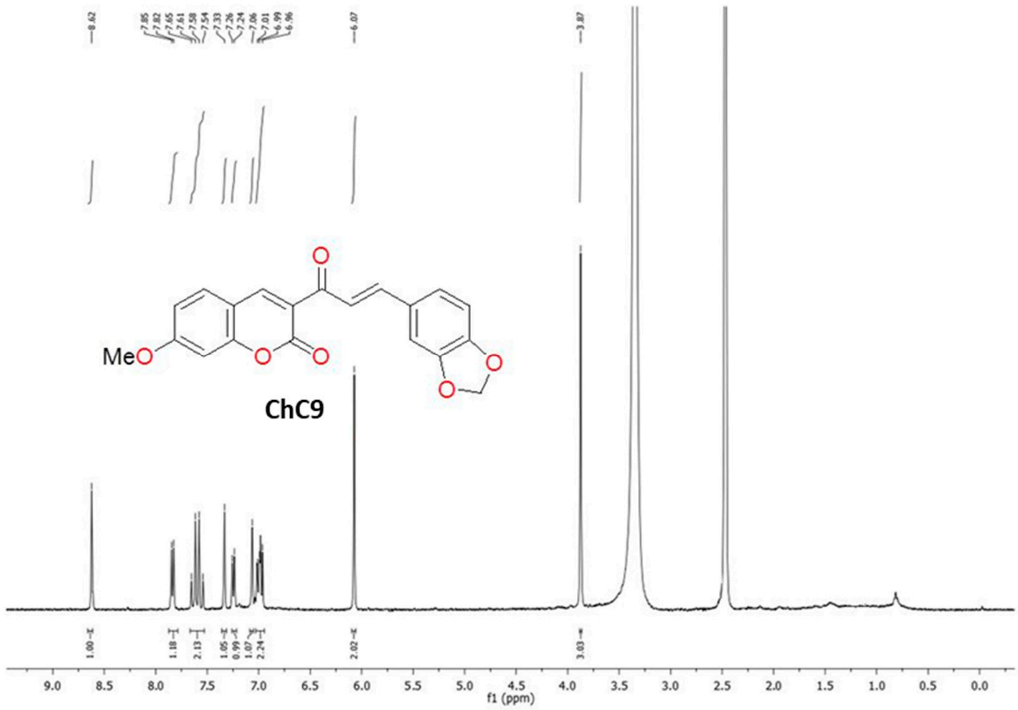


**Figure S10.** $^{1}$H NMR spectrum of **ChC9**.

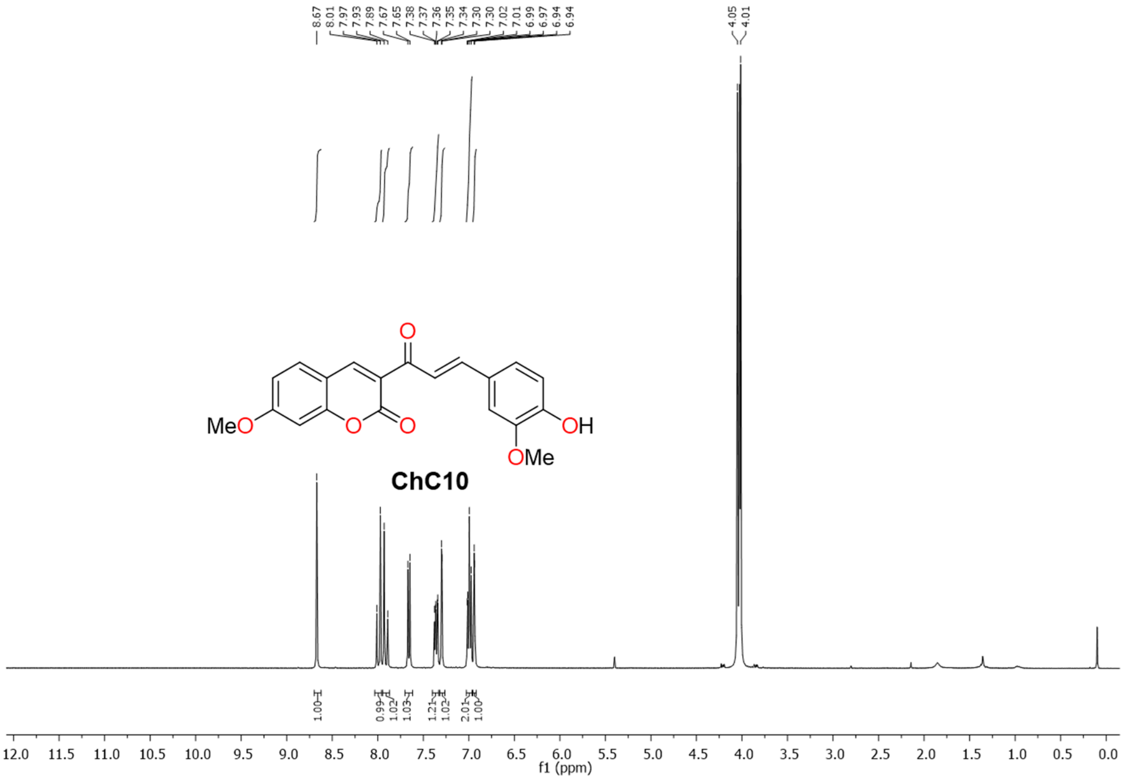


**Figure S11**. $^1$H NMR spectrum of **ChC10**.

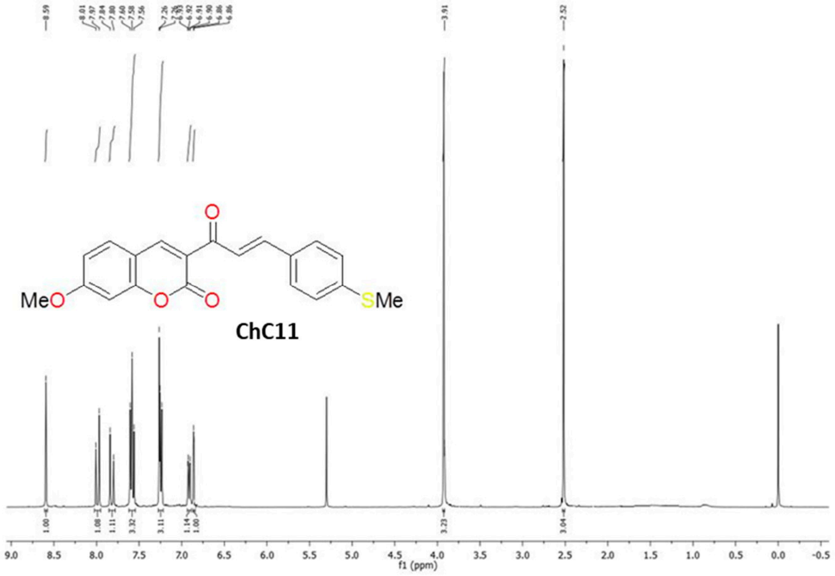


**Figure S12**. $^1$H NMR spectrum of **ChC11**.

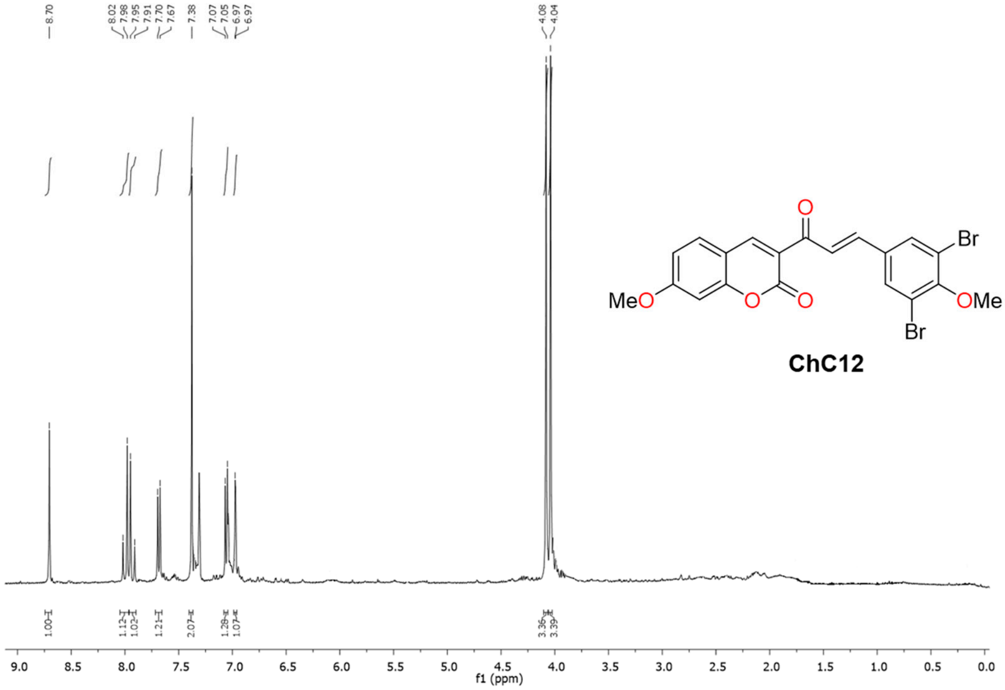

**Figure S13**. $^{1}$H NMR spectrum of **ChC12**.

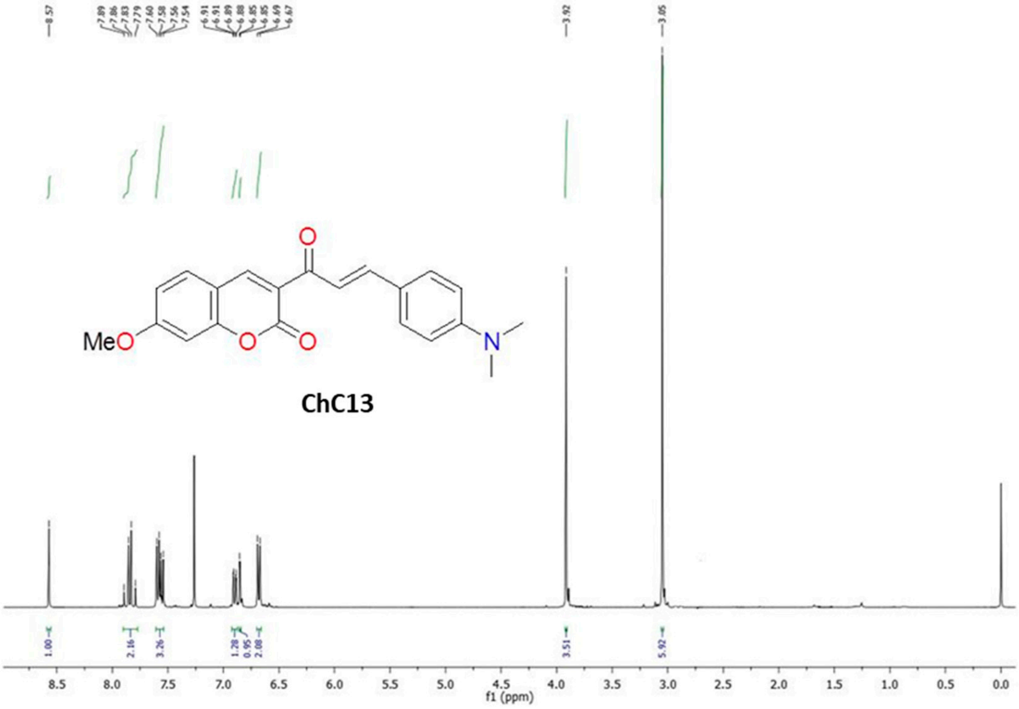

**Figure S14**. $^{1}$H NMR spectrum of **ChC13**.

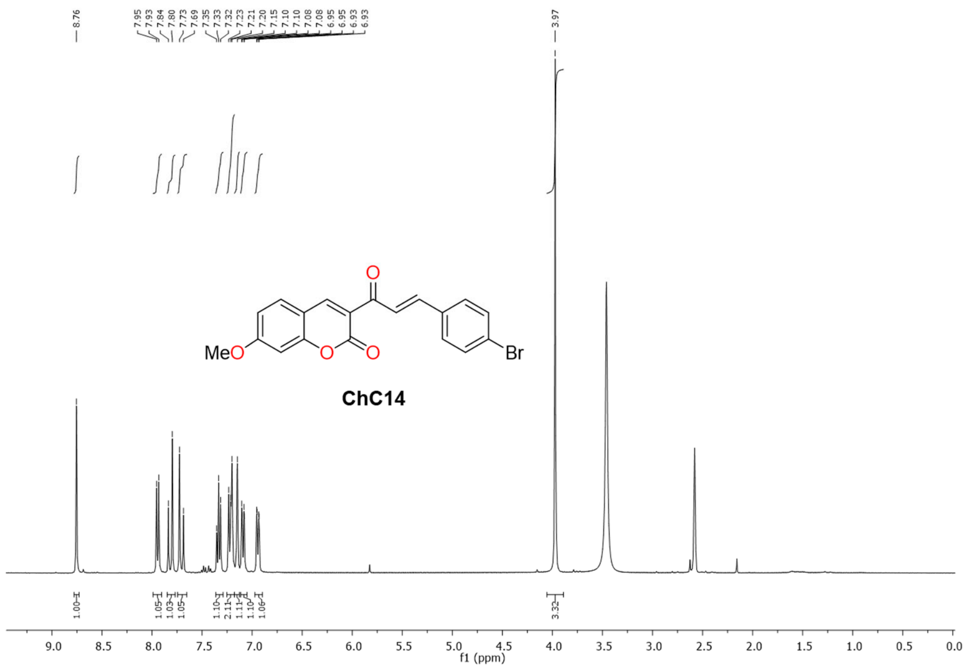


**Figure S15**. $^1$H NMR spectrum of **ChC14**.

*Homology modeling analysis.*

In the current study, the three-dimensional rMAO-B model was built by homology with the hMAO-B with which it displays a sequence identity of 86.57% and a similarity of 89.4%, see Figure 1. The stereochemical evaluation of backbone psi and phi dihedral angles of the rMAO-B model gave 95.3% in the most favorable region, and 4.0% in the additional allowed region, proving to be acceptable (Table 3 and Figure 2). In general, a score close to 100% indicates the good stereo-chemical quality of the model [29]. The total quality G-factor of -0.12 for the rMAO-B model was also a sign of good quality (acceptable values of the G-factor in PROCHECK are between 0 and −0.5, with the best models displaying values close to zero). The rMAO-B model is therefore acceptable with respect to parameters derived from high-resolution protein structures, and no bad contacts or scores are evident for the main chain or side chain parameters. Therefore, these PROCHECK results suggest that the predicted model was of good quality.

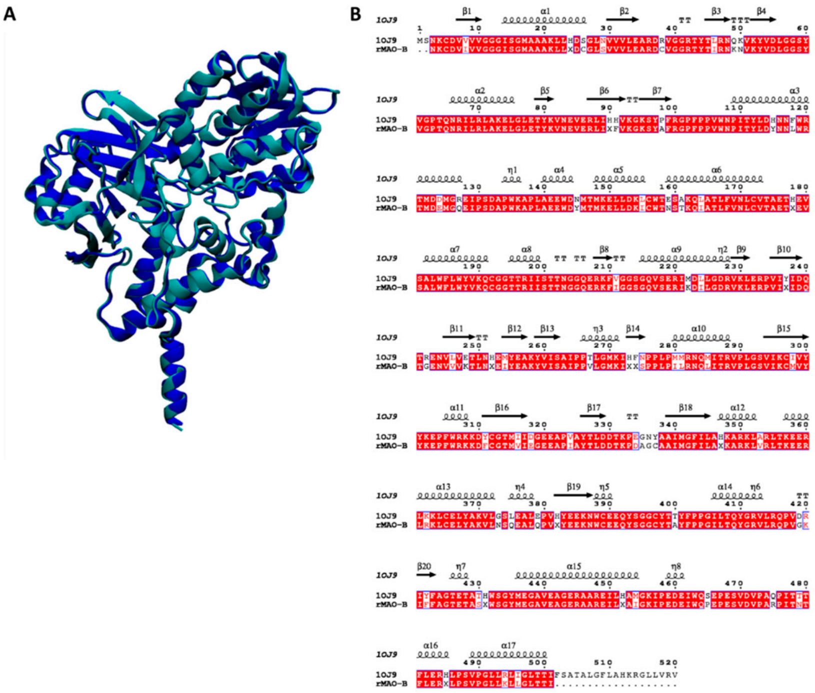


**Figure S16**. A) Superimposed hMAO-B (PDB code 1OJ9) template (cyan) on the rMAO-B homology model (blue). B) Alignment generated by the ESPript 3.0 webtool [30] of hMAO-B and the rMAO-B model. Percent identity for the rMAO-B sequence was 86.5%.

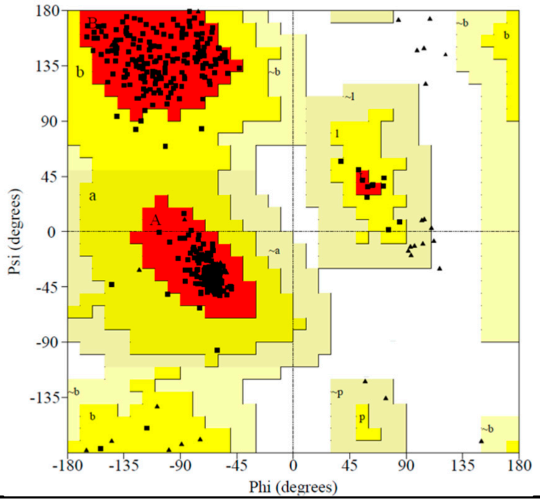


| | Ramachandran Plot Quality (%) | | | | Goodness Factor | | |
|---|---|---|---|---|---|---|---|
| | Most Favored | Additional Allowed | Generously Allowed | Dis-Allowed | Dihedral | Covalent | Overall |
| rMAO-B model | 95.3 | 4.0 | 0.0 | 0.7 | -0.07 | -0.22 | -0.12 |

**Figure S17**. Ramachandran plot generated via PROCHECK for the rMAO-B model. PROCHECK shows the residues in most favored (red), additionally allowed (yellow), generously allowed (pale yellow) and disallowed regions (white color).

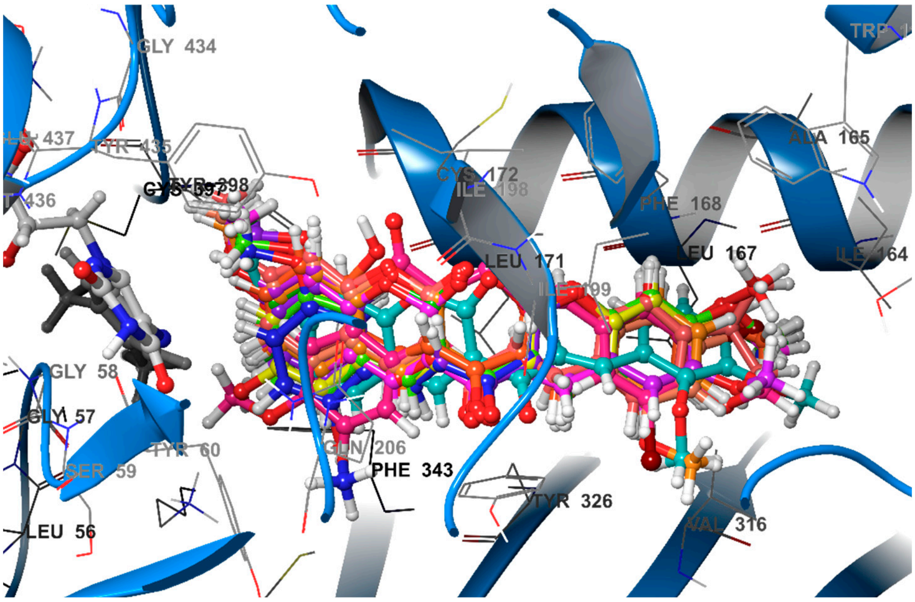


**Figure S18.** Alignment of all the ChC ligands docked in complex with rMAO-B.
